# The Effects of a Modified Mediterranean Diet on Gut Microbiota and Chemotherapy Side Effects in Patients With Metastatic Colorectal Cancer Undergoing First-Line Chemotherapy With or Without Either Antiepidermal Growth Factor Receptor or Antivascular Endothelial Growth Factor Agent: Protocol for a Randomized Pilot Study in Italy

**DOI:** 10.2196/72950

**Published:** 2025-11-14

**Authors:** Salvatore Artale, Francesca Filiali, Elena Beretta, Francesca Arosio, Federica Cazzaniga, Carlo Tersalvi, Michele Sofia, Paola Tagliabue, Paola Pozzi, Andrea Colombo, Claudia Carbone, Lisa Pietrogiovanna, Magda Verga, Paola Nova, Rossella Calori, Rossella Renso, Selene Rota, Stefania Aglione, Italo Manfrida, Giovanna Facendola, Alessandra Trojani, Maria Chiara Dazzani, Sabrina Basciani, Maria Grazia Valsecchi, Giulia Capitoli, Cinzia Cocola, Clarissa Consolandi

**Affiliations:** 1 Oncology Unit Azienda Socio-Sanitaria Territoriale della Brianza Vimercate Hospital Vimercate Italy; 2 Oncology Unit Azienda Socio-Sanitaria Territoriale della Brianza Desio Hospital Desio Italy; 3 General Management Azienda Socio-Sanitaria Territoriale della Brianza Vimercate Hospital Vimercate Italy; 4 Health Management Department Azienda Socio-Sanitaria Territoriale della Brianza Vimercate Hospital Vimercate Italy; 5 Department of Hematology and Oncology Azienda Socio-Sanitaria Territoriale Grande Ospedale Metropolitano Niguarda Milan Italy; 6 Department of Experimental Medicine Sapienza University of Rome Rome Italy; 7 School of Medicine and Surgery Center of Bioinformatics, Biostatistics and Bioimaging University of Milano-Bicocca Monza Italy; 8 Institute of Biomedical Technologies National Research Council Segrate Italy

**Keywords:** colorectal cancer, modified mediterranean diet, gut microbiota, metagenomics, metabolome, pilot study

## Abstract

**Background:**

The gut microbiota is attracting increasing interest as a factor possibly impacting colorectal cancer risk, therapy toxicity, and, as a consequence, patient’s quality of life. It has been observed that microbial imbalance in the gut and in cancer tissue is facilitated by a Western type of diet, rich in meat, sugars, and refined grains, while a Mediterranean diet, rich in low saturated fat and fibers, promotes gut eubiosis, and results in reduced risk of developing colorectal cancer. Specifically, a high fiber content diet has been associated with a reduced incidence of therapy related adverse events in patients with malignant melanoma.

**Objective:**

This study aimed to analyze and compare the gut microbiota of patients with metastatic colorectal cancer undergoing first-line chemotherapy with or without a biological agent (antiepidermal growth factor receptor or antivascular endothelial growth factor), and receiving either a free standard Western diet, or a modified Mediterranean diet, and the impact of microbiota on chemotherapy toxicity.

**Methods:**

This is a pilot nondrug, interventional prospective, randomized, controlled, single-center (Italian), open-label trial. Patients (n=40) living in Italy, and with a local style of life, will be randomized 1:1 to either a modified Mediterranean diet or a free Western-type diet. Blood and fecal samples will be collected at baseline and control visits, for metagenomic and metabolomic analysis. The primary endpoint is the Firmicutes:Bacteroidetes ratio after completion of the third cycle of first-line chemotherapy (time T1). Secondary endpoints are (1) the percentage of patients experiencing gastrointestinal side effects at T1, (2) the percentage of patients experiencing grade 3/4 gastrointestinal side effects at T1, and (3) changes in the Firmicutes:Bacteroidetes ratio, overall microbiome composition, and metabolome at T1, and after the sixth chemotherapy cycle (T2) versus baseline.

**Results:**

This pilot trial received ethics approval on July 24, 2024. By July 2025, a total of 17 participants have been recruited. The study will conclude with the visit at T2 for the last enrolled patient. Results are expected to be published in October 2028.

**Conclusions:**

This study has the potential to provide critical insights into the role of diet in modifying the gut microbiota, diminishing chemotherapy-related side effects, and possibly enhancing the therapeutic efficacy in metastatic colorectal cancer by improving tolerability. In addition, data may pave the way for future research in immunotherapy, potentially influencing both clinical practice and public health strategies.

**Trial Registration:**

Clinicaltrial.gov NCT06794931; https://clinicaltrials.gov/search?term=NCT06794931

**International Registered Report Identifier (IRRID):**

DERR1-10.2196/72950

## Introduction

Colorectal cancer (CRC) is one of the most frequent and deadly malignant tumors, globally [1]. The overall incidence and mortality rates are increasing, probably due to population aging, and increasing incidence of CRC risk factors associated with socioeconomic development [1]. In addition, a relevant rise in early-onset CRC diagnosis was observed in the last three decades [2].

Among the risk factors for CRC, some are unmodifiable, such as age, sex, inflammatory bowel disease, and genetic predisposition [3,4], while others are controllable, such as overweight or obese body weight, smoking, sedentary habits, and unhealthy diet [4,5]. Several of these determinants interact with the gut microbiota (GM), which represents the collection of microorganisms (ie, bacteria, viruses, fungi, and archaea) colonizing the gastrointestinal tract. Therefore, GM attracts increasing interest as an initiator of CRC [6].

The main representatives of the GM are the Firmicutes, Bacteroidetes, Actinobacteria, and Proteobacteria phyla, with variable prevalence along the oxygen gradient in the lumen of the gastrointestinal tract [7]. These microorganisms, when in equilibrium (a condition mentioned as eubiosis), are beneficial to the host by many activities including fermenting complex carbohydrates to short chain fatty acids (SCFAs), that are used as energy source by gastrointestinal epithelial cells [8]. In addition, the eubiotic GM contributes to the synthesis of vitamins [9], protection from pathogen colonization [10], and maintaining mucosal integrity [11]. When the microbial balance is perturbed by any cause (a condition referred to as dysbiosis), the gut barrier is disrupted, microbial invasiveness increases, and commensal bacteria may turn to pathogens, resulting in inflammation and activation of tumorigenic pathways [11].

Specific changes in the GM have been associated with steps in CRC progression, suggesting that dysbiosis may contribute to such disease states [12]. The lumen- or mucosa-associated GM in CRC patients has higher proportions of *Fusobacterium nucleatum*, *Bacteroides fragilis*, and *Escherichia coli* species*,* compared to the GM of healthy individuals [13-15].

It is known that the GM has a role as a modulator of the efficacy and toxicity of conventional chemotherapeutic agents used for CRC treatment [16]; the presence of certain bacterial species can exacerbate chemotherapy-induced side effects and pharmacological therapy toxicity, but conversely, microbiota can also enhance treatment efficacy [17].

The microorganisms act on tumor microenvironment through many mechanisms, including genotoxin and free radical production, modification of signaling pathways in host cells, immune modulation, interaction with drug metabolism, and synthesis of small molecules, lipids, proteins, sugars, terpenoids, and organic acids, which may contribute to the pathogenesis of cancer [12,18].

Not only the GM affect the metabolism in the gut, but also compounds and metabolites may influence the GM contributing to the development of a dysbiotic state. It has been repeatedly observed that the presence of *F. nucleatum* in the gut and in CRC tissue is facilitated by a Western type of diet, rich in meat, sugars, and refined grains [14,19], generating intestinal microbial imbalance. On the contrary, the Mediterranean diet, rich in low saturated fat and fibers, promotes eubiosis, and results in reduced risk of developing CRC [20-23]. Epidemiological data show that a healthy diet and eubiosis contribute to reduced risk of CRC [23], while an unhealthy diet is associated with enhanced risk, recurrence, and mortality of CRC [21,23,24]. However, the exact mechanisms through which diet affects CRC are still incompletely understood.

Consuming fiber- and phytochemical-rich foods promotes an increase of beneficial bacterial populations capable of preventing mucus layer damage, reducing inflammation, and lowering CRC risk [14]. It has been observed that in *F. nucleatum*-positive patients, a Mediterranean-type diet, rich in fibers and whole-grains, referred to as a “prudent diet,” can reduce CRC onset [14].

Overall, current evidence suggests that acting on GM composition by a dietary intervention may be a suitable strategy to prevent CRC risk, can improve outcomes of therapy, and reduce toxicity in CRC patients. GM biodiversity can be improved by the introduction of microbiota-accessible carbohydrates, which are nondigestible by the host, but fermentable by gut bacteria [25]. Microbiota-accessible carbohydrates, classified as prebiotics, are selectively used by microorganisms as substrates to produce SCFAs, such as acetate, propionate, and butyrate [26,27]. Thus, they provide energy for colonocytes, regulate mucin (MUC2) expression, which is critical for intestinal barrier formation, and support immune function. Common prebiotics such as galacto-oligosaccharides and fructo-oligosaccharides, stimulate the growth of beneficial Bifidobacteria and Lactobacilli, modulate cytokine activity, and suppress proinflammatory markers, while promoting apoptosis, and protecting the mucosal barrier [28,29]. Additionally, postbiotics, metabolites produced during bacterial fermentation of nondigestible compounds, mediate the GM-host interaction and facilitate the synthesis and regulation of mucin [30].

Relevant to the ideation of this study, recent data from the Diet study on patients with metastatic melanoma, suggest that a high fiber content diet is associated with a reduction in therapy associated adverse events [31].

Firmicutes are the main producers of butyrate, the preferred metabolic substrate for colon epithelial cells, while acetate and propionate, which contribute to barrier protection, are predominantly derived from Bacteroidetes [32]. In vitro, butyrate administration enhances mucin protein content, supporting probiotic adhesion and inhibiting *E. coli* pathogenicity [32]. Butyrate also regulates mucin expression and epithelial tight junction proteins, strengthening the intestinal barrier [33].

Another beneficial compound acquired through the diet is tryptophan, an essential amino acid that maintains mucosal homeostasis via bacterial metabolism. A protein-rich diet with tryptophan may enhance intestinal defenses [34].

Overall, nutritional interventions, including the use of prebiotics and probiotics, appear capable of modulating the GM, promoting eubiosis, stimulating the production of SCFAs, resulting in enhanced mucus layer, barrier function and prevention of chronic inflammation. Possibly, changes in the GM could result in reduced side effects of chemotherapy, and improved quality of life. Based on this evidence, and investigating these hypotheses, a pilot study was designed to determine and compare the GM of patients with metastatic CRC undergoing first-line chemotherapy with or without a biological agent (antiepidermal growth factor receptor [EGFR] or antivascular endothelial growth factor [VEGF]), and receiving, based on random allocation, either a free choice Standard Western Diet (SWD), or a Modified Mediterranean Diet (MMD).

## Methods

### Ethical Considerations

This pilot trial has received ethics approval from the Ethics Committee Lombardia 2, Milan, Italy (reference n. R1926/24 – L2-130). All participants will provide full written informed consent before enrolling in the study. The processing of personal data collected during the study complies with the General Data Protection Regulation (GDPR), according to legislative decree. 196/2003, and any Italian laws governing personal data protection. The data of the enrolled participants will be kept strictly confidential and will be pseudo-anonymized. The trial will be conducted in conformity with the Declaration of Helsinki and Good Clinical Practice guidelines.

### Study Design

**Figure 1:**
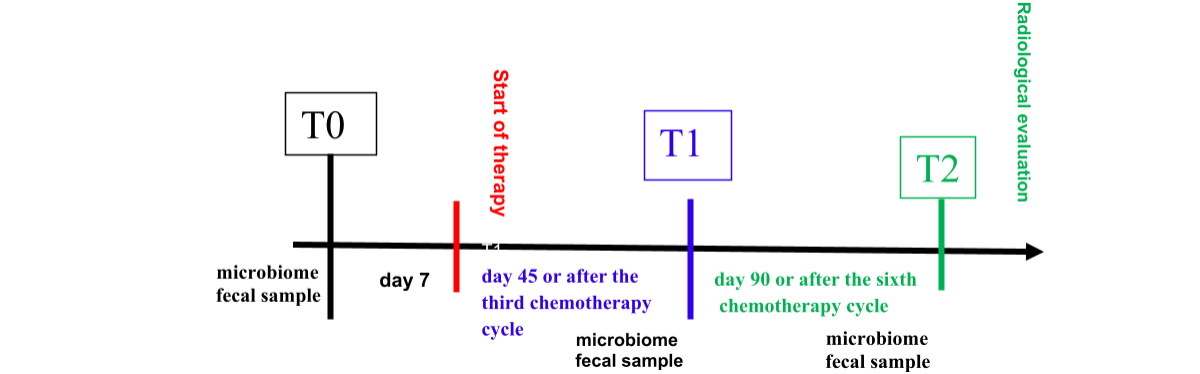
Study timepoints.

This is a pilot nondrug interventional clinical study, involving biological sampling, designed as a prospective, randomized, and controlled, single-center, Italian, open-label trial with a 1:1 allocation ratio (Clinicaltrial.gov NCT06794931). Figure 1 shows the workflow of MIC-1. The study will be conducted in Vimercate Hospital, Azienda socio sanitaria territoriale (social and health territorial unit) della Brianza, 20871 Vimercate, Italy. The randomization lists will follow a block design generated by dedicated software and maintained by an independent data manager. Stratification is based on gender. Recruitment of participants began in October 2024, and it is expected to be completed in October 2027.

Given the relevance to study results of patients’ diet habits, only subjects living in Italy, and with a local style of life (as assessed by a free interview during the first visit) will be enrolled, to reduce variability in the control group. In addition, homogeneous performance status was necessary in the patients’ sample. The nutritional state will be assessed before enrollment to exclude patients with malnutrition.

The inclusion criteria are listed in Textbox 1.

Malnutrition Universal Screening Tool is a five-step screening tool planned to identify adults, who are malnourished, at risk of malnutrition (undernutrition), or obese, and including also management guidelines for a care plan [35].

Participants will not be eligible if they meet any of the exclusion criteria (Textbox 2)

**Inclusion criteria.**

**Inclusion criteria.**

- Italian residence and habits;
- Eastern Cooperative Oncology Group performance status: 0 or 1;
- age at diagnosis should be 18 years or older;
- histologically and radiologically confirmed diagnosis of metastatic CRC based on Response Evaluation Criteria in Solid Tumors 1.1 criteria;
- first-line chemotherapy with or without a biological agent (anti-EGFR or anti-VEGF);
- Malnutrition Universal Screening Tool score: 0-1;
- written informed consent.

**Exclusion criteria.**

**Exclusion criteria**

- living outside Italy;
- age<18 years;
- previous oncological medical treatments;
- indication for parenteral and/or enteral nutrition;
- Malnutrition Universal Screening Tool Score: ≥2.

### Intervention

#### Study Arms: Arm A: MMD; Arm B: SWD (designed according to patient's habits)

Patients will be randomly included in one arm according to a block diagram generated by dedicated software and stored by a study independent data manager.

#### Diet

The authors planned the present diet on the bas`is of their previous experience [36]. An exemplary diet is presented in Table 1. To develop a nutritional plan favoring bacterial biodiversity, we are mainly focusing on foods containing high-quality prebiotic fibers, reducing the total amount of the fiber content compared to standard guidelines (Livelli di Assunzione di Riferimento di Nutrienti [Nutrient Intake Levels]-Italian Dietary Reference Intakes) to limit the gastrointestinal side effects associated with chemotherapy. To this aim, foods containing resistant starch are preferred. Evidence indicates that such starch promotes changes in bacterial abundance, specifically increasing Firmicutes and particularly *Faecalibacterium prausnitzii*, and the production of butyrate, a SCFA essential for intestinal health [37,38].

Moreover, the dietary plan incorporates foods rich in polyphenols such as brown rice, extra virgin olive oil, and green tea, which have been shown to positively influence cellular aging, blood cholesterol levels, and apoptosis [39]. Polyphenols have been demonstrated to increase protective bacteria (Bifidobacteria and Lactobacilli) and butyrate-producing bacteria (*Faecalibacterium prausnitzii*). Furthermore, polyphenols are known to reduce pathogenic bacteria, including Proteobacteria like *E coli* [37,38].Beta-glucans, indigestible polysaccharides found in barley, oats, and mushrooms, are included in the MMD since GM bacteria metabolize these compounds to produce SCFAs, which are crucial for the nutrition and integrity of intestinal mucosal cells.

**Table 1:** Exemplary MMD; changes may be planned according to patient’s requests. Each lunch and each dinner must be represented in a week, but the sequence within the 7 days may be adjusted by the patient. Each meal may be added with 3 spoons of extra virgin olive oil (evoo). Admitted vegetables: steamed zucchini, carrots, fennels, asparagus, artichokes, chard, spinach, cauliflower; raw or baked Belgian endive; pan fried mushrooms; salads; tomato sauce. Admitted fish: cod, sole, hake, sea bass, bream. Drink at least 2 l water per day.

	Breakfast	Snacks	Lunch	Dinner
Monday	Either: Black coffee or tea with stevia or mannitol; 50 g rye bread; 50 g lactose free ricotta or 2 teaspoons of evoo, Or: 125 g lactose free yogurt with 3 spoons of oat bran	Either: one fresh fruit without peel, Or: 150 g kefir, Or: 10-12 olives, Or: 30 g crackers	80 g whole rice, 100 g fish, 200 g of admitted vegetables	3 fried egg whites with 200 g mushrooms and Parmigiano cheese; 80 g hard wheat bread
Tuesday	As Monday	As Monday	Vegetable soup with 100 g beans + 50 g pearl barley + 2spoons of Parmigiano cheese	200 g of baked or steamed admitted fish or 160 g chicken fillet, 200 g of admitted vegetables, 80 g toasted hard wheat bread
Wednesday	As Monday	As Monday	80 g basmati rice, 80 g Emmenthal cheese, 200 g of admitted vegetables	2 boiled eggs, 200g admitted vegetables, 80 g of hard wheat bread
Thursday	As Monday	As Monday	90 g (weighted before cooking) lentils, 200 g of admitted vegetables, 40 g of toasted hard wheat bread	200 g roasted, baked, or steamed admitted fish, 200 g admitted vegetables, 80 g toasted hard wheat bread
Friday	As Monday	As Monday	3 fried egg whites with 200 g steamed zucchini and evoo (or one egg and 2 egg whites with mushrooms), and 80 g of toasted hard wheat bread	Chickpeas or beans (90 g) cream, 200 g admitted vegetables, 40 g toasted hard wheat bread
Saturday	As Monday	As Monday	80 g semolina pasta with 150 g artichokes and 50 lactose free ricotta; Or: 60 g semolina pasta with 150 g cooked beans, and 150 g salad	Cream with 100g topinambur, vegetable broth, 1 potato, and evoo + 20 g hard wheat bread + 150 baked blue fish and 150 g admitted vegetables
Sunday	As Monday	As Monday	80 g semolina pasta with tomato sauce, 50 Parmigiano cheese, 200 g admitted vegetables	150 g roasted salmon or 180 g trout; 200 g admitted vegetables; 80 g of toasted hard wheat bread

Fermented products with low lactose content, such as kefir, are incorporated due to the proven effects on enhancing GM biodiversity, and on modulating immune function by supporting specific beneficial bacterial populations [40].

Highly antioxidant foods, such as fruits, vegetables, whole grains, and legumes, are useful to increase the abundance of *Akkermansia Muciniphila*, a key bacterium associated with maintaining a healthy intestinal barrier, and reducing inflammation mediated by lipopolysaccharides [41].

The plan also incorporates high-quality protein sources that also exhibit prebiotic properties, such as ricotta cheese (from cow, sheep, or goat milk, lactose-free if necessary). These proteins have been shown to support symbiotic bacteria and promote intestinal health [42].

The inclusion of salmon and other fish (eg, mackerel and sardines) rich in omega-3 fatty acids aims to enhance the growth of butyrate-producing bacteria, and reduce inflammation, supporting the gut's overall health [43].

#### Study Workflow

During a routine control visit, eligible patients are informed about the study and, upon signing the written informed consent, undergo nutritional screening containing the components shown in Textbox 3, Table 2, and Figure 1.

Finally, patients are provided with a container for collecting baseline fecal samples.

After the nutritional assessment confirms eligibility, patients will attend baseline visit (time T0) to be randomized into study arms.

Follow-up visits will align with the patient's chemotherapy schedule. Visits will occur after the third chemotherapy cycle (approximately at day 45 – time T1), and the sixth chemotherapy cycle (approximately at day 90 – time T2).

At each visit (T0, T1, T2), patients will return a fecal sample and receive a container for further sampling. In addition, they will be administered the 30-item European Organization for Research and Treatment of Cancer quality of life questionnaire. At T1 and T2, patients will undergo the activities listed in Textbox 4.

**Components of the nutritional screening.**

- A detailed nutritional assessment using a dedicated chart,
- Routine blood tests,
- Assessment of serum carcinoembryonic antigen and carbohydrate antigen 19-9 levels,
- Serum sampling for nutritional markers (C reactive protein, prealbumin, albumin, interleukin [IL]-6, transferrin),
- Assessment of lifestyle factors (ie, sleep quality, smoking habits),
- Recording of a 24-hour dietary recall (food diary of the past 24 hours),
- Anthropometric measurements (body weight, height, BMI, and bioimpedance analysis),
- Calculation of caloric-protein needs,
- Malnutrition Universal Screening Tool score.

**Table 2:** Study workflow.

	T0: baseline visit	T1: day 45 or after the third chemotherapy cycle	T2: day 90 or after the sixth chemotherapy cycle
Clinical interventions	Informed consent Recording of lifestyle Nutritional assessment Blood tests CEA^a^ and CA19.9^b^ Food diary Anthropometric measurements Caloric protein needs are calculated MUST^c^ score EORTC QLQ-C30^d^ test Fecal sample container is received	Nutritional assessment Gastrointestinal events report Blood tests CEA and CA19.9 Oncologic visit EORTC QLQ-C30 test Fecal sample is given and a new container is received KIDMED^e^ test	Nutritional assessment Gastrointestinal events report Blood tests CEA and CA19.9 Oncologic visit EORTC QLQ-C30 test Fecal sample is given KIDMED test Routine radiological evaluation
Metagenomic and metabolomic analysis	—^f^	On frozen fecal sample	On frozen fecal sample
Firmicutes:Bacteroidetes ratio	—	Assessed from OTU^g^ tables	Assessed from OTU tables

^a^CEA: carcinoembryonic antigen.

^b^CA19.9: carbohydrate antigen 19-9.

^c^MUST: malnutrition universal screening tool.

^d^EORTC QLQ-C30: European Organization for Research and Treatment of Cancer quality of life questionnaire.

^e^KIDMED: Mediterranean diet quality index for children and adolescents.

^f^Not applicable.

^g^OTU: operational taxonomic unit.

**Activities at T1 and T2.**

- Nutritional assessment to evaluate compliance with the assigned diet,
- Routine blood tests,
- Assessment of carcinoembryonic antigen and carbohydrate antigen 19-9,
- Serum analysis for nutritional markers (C reactive protein, prealbumin, albumin, IL-6, transferrin),
- Clinical examinations as required by the oncological treatment plan,
- A modified adherence questionnaire derived from the Mediterranean Diet Quality Index for Children and Adolescents (KIDMED) test to assess dietary compliance [44,45],
- Recording of reported gastrointestinal events

At T2, a radiological evaluation as per routine clinical practice is also planned.

To assess chemotherapy side effects, gastrointestinal events will be considered and the proportions of subjects with gastrointestinal events (diarrhea, nausea, and vomit) and with sever gastrointestinal events will be compared in the 2 groups of patients 45 days after beginning chemotherapy with or without the biologic agent. Events will be scored by the National Institutes of Health or National Cancer Institute Common Terminology Criteria for Adverse Events criteria, version 5.0 [46].

#### Adherence to the Diet

The KIDMED (Mediterranean Diet Quality Index for Children and Adolescents) test is designed to evaluate the adherence to the Mediterranean diet of children and young people [44]. As the diet included in this study introduces several changes to the Mediterranean diet and excludes dairy foods to limit the risk of diarrhea, the KIDMED test will be changed accordingly, based on previously published experience [36,45].

#### Metagenomic and Metabolomic Analysis

Frozen fecal samples will be sent to the Institute of Biomedical Technologies, National Research Council (Segrate, Milan, Italy) for metagenomic and metabolomic analysis.

### Endpoints

The primary objective of this pilot study is to evaluate the impact of the MMD compared to the SWD, on GM biodiversity, in patients with metastatic CRC undergoing first-line chemotherapy with or without a biological agent (anti-EGFR or anti-VEGF).

To this aim, the primary endpoint is the Firmicutes:Bacteroidetes ratio after completion of the third cycle (T1) of first-line chemotherapy, which will be compared between the 2 randomized arms.

Secondary objectives are to assess the impact of the MMD versus the SWD on tolerability of chemotherapy.

To these goals, secondary endpoints are: (1) the percentage of patients experiencing gastrointestinal side effects (diarrhea, nausea, and vomiting) at T1; (2) the percentage of patients experiencing severe gastrointestinal side effects (Grade 3/4 diarrhea, nausea, or vomiting, according to Common Terminology Criteria for Adverse Events criteria) at T1; (3) changes in the Firmicutes:Bacteroidetes ratio, overall microbiome composition through alpha-diversity (Shannon index) and beta-diversity (Weighted Unifrac) calculation to obtain information about richness, evenness, phylogenetic relationships, and relative abundance and metabolome at T1 and T2 versus baseline.

Exploratory objective is to assess the impact of the MMD versus the SWD on the quality of life.

### Screening Methodology for Metagenomic and Metabolomic Analysis

#### DNA Extraction Techniques, 16S rRNA Gene Metabarcoding Library Preparation, and Ultramassive Sequencing

Fecal samples, collected from study patients, are stored at –-80°C until DNA extraction, carried out by Institute of Biomedical Technologies, National Research Council. Automated bacterial DNA isolation will be performed by Maxwell RSC Fecal Microbiome DNA Kit (Promega, Milan, Italy) and DNA quality will be assessed using the TapeStation 4200 system (Agilent Technologies). Only samples with a DNA integrity number ≥ 4 will proceed to further analyses. Samples with DNA integrity number <4 at T1 will result in the patient’s exclusion from the study, and previously collected data will not be used. Metabarcoding libraries will be prepared following the “16S Metagenomic Sequencing Library Preparation” protocol, which includes amplification of the V3 and V4 hypervariable regions of the 16S rRNA gene and indexing with DNA or RNA UD Indexes (Illumina).

Libraries will be quantified using the Qubit 4 fluorometer (Invitrogen) and pooled at equimolar concentrations. Sequencing will be performed on an Illumina MiSeq or NextSeq1000 platform, by paired-end 2×300 bp runs.

#### Bioinformatics Analysis of Sequencing Data

Raw reads derived from sequencing will be processed using a custom bioinformatics pipeline. Overlapping paired reads will be assembled into a single fragment using PandaSeq software (publicly available) [47]. Taxonomic units will be generated using the zero-radius operational taxonomic unit (OTU) method and classification. Classification will use the QIIME suite [48], unoise3 algorithm [49], RDP Classifier [50], and the SILVA 16S rRNA database [51].

Alpha-diversity will be calculated using metrics such as Chao1, Shannon Index, Observed Species, Good’s Coverage, and Faith’s Phylogenetic Distance. Beta-diversity will be assessed with weighted and unweighted UniFrac metrics, coupled with Principal Coordinate Analysis. Genus-specific reads will be further classified to species level via Basic Local Alignment Search Tool realignment against a custom database derived from NIH-NCBI sequences [52]. Metabolic pathways will be inferred using Phylogenetic Investigation of Communities by Reconstruction of Unobserved States software and the Kyoto Encyclopedia of Genes and Genomes pathway database.

#### Shannon Diversity Calculation

The Shannon diversity index will be calculated from OTU tables containing absolute counts at the species level using the vegan R package (v2.5-6). Species with an average relative abundance of < 0.1% across all samples will be excluded. OTU counts will be normalized via rarefaction analysis before diversity calculations.

#### Firmicutes:Bacteroidetes Ratio Evaluation

The Firmicutes/Bacteroidetes ratio will be derived from OTU tables grouped at the phylum level. Phyla with an average relative abundance of <0.1% across all samples will be excluded.

### Metabolomic Analysis

Metabolites will be extracted by preparing aqueous fecal extracts by TissueLyser II homogenizer (Qiagen, Hilden, Germany). Samples will be subsequently derivatized with pentafluorobenzyl bromide in acetone, and SCFAs (acetate, propionate, butyrate) will be isolated into the organic phase using hexane (all chemicals sourced from Sigma-Aldrich, St. Louis, MO, USA). Samples will be analyzed by a 5977B single-quadrupole mass spectrometer coupled to an Intuvo 9000 gas chromatograph (Agilent Technologies, Santa Clara, CA, USA). Chromatographic separation will be conducted using a DB-FATWAX UI column (30 m × 0.25 mm × 0.25 µm; Agilent Technologies), and detection will be achieved via single ion monitoring for enhanced sensitivity. Absolute quantification of SCFAs (acetate, propionate, butyrate) will be achieved using calibration curves from standard solutions. Obtained data will be analyzed using MassHunter Quantitative software (version 10.2, Agilent Technologies). Furthermore, metabolite concentrations will be correlated with bacterial composition by calculating Spearman correlation coefficients between metabolites and bacterial genera present at ≥ 1% in at least one sample. Only correlations with *P*<.05 will be considered statistically significant.

### Rationale for the MMD Plan for Arm A Patients

The diet assigned to each patient will be strictly personalized, considering sex, food preferences, anthropometric characteristics, underlying oncological condition, clinical characteristics (eg, comorbidities), and pharmacological treatment plan.

Daily caloric needs will be calculated by multiplying the basal metabolic rate (determined using the Harris and Benedict formula) by 1.3, assuming moderate physical activity.

### Sample Size and Statistical Analysis

The enrollment of 16 patients per arm is required to show a relative reduction of 35% in the Firmicutes:Bacteroidetes ratio from 40% (SD 13) in arm SWD (control arm) to 26% in Arm DMM at time T1, with a significance level of the Mann-Whitney test of 5% (two-sided) and a power of 80%. To consider the possibility of approximately 20% dropouts, 20 patients per arm will be randomized.

The primary comparison of the Firmicutes:Bacteroidetes ratio in Arm DMM vs SWD at T1, will be done with the Mann-Whitney test; an additional analysis adjusting for potential confounding variables (eg, age, comorbidities, chemotherapy type) will be done using quantile regression with these factors as covariates.

For secondary endpoints involving the comparison of the percentage of patients with gastrointestinal effects between the 2 arms, the chi-Square test will be applied.

To adjust for potential confounding factors, an additional analysis will be performed with a logistic regression model.

The analysis of secondary endpoints on changes in the Firmicutes:Bacteroidetes ratio at T1 and T2 versus baseline will be done with the Wilcoxon Signed-Rank test.

The primary and secondary endpoint analyses will follow the Intention-to-Treat principle.

After describing the KIDMED score distribution, expressing the level of adherence to the MMD, an additional per-protocol analysis will be performed excluding patients with a poor adherence, defined as KIDMED test score ≤3 [45].

The sample size is underpowered to draw definitive conclusions on response rates, toxicity, or quality of life differences; clinical outcomes are exploratory and meant to generate hypotheses for future properly powered studies.

Subgroup analyses within different chemotherapy regimens will be performed for exploratory purposes only, due to the relatively limited number of patients, with the aim of having indications on the possible different efficacy and safety of the MMD vs SWD and of proposing additional in-depth studies.

The significance level for all tests is set at 5% (2-tailed).

## Results

This pilot trial has received ethics approval from the Ethics Committee Lombardia 2, Milan, Italy (reference R1926/24 – L2-130), on July 24, 2025. Recruitment started in October 2024 and will be completed in 36 months. Data collection commenced in October 2024, with the enrollment of the first patient. By July 2025, a total of 17 participants have been recruited. The study will conclude with the visit at T2 for the last enrolled patient. Results are expected to be published in January 2028.

## Discussion

### Expected Findings and Perspectives

The main finding expected from this pilot study on patients with metastatic CRC undergoing first-line chemotherapy with or without anti-EGFR or anti-VEGF agents would be a significant difference in GM between patients following a predominantly plant-based MMD, and those following a free Western diet. To this purpose, our research project aims to compare the effects of the Mediterranean diet, characterized by the exclusion of sucralose, red meats, and processed meats (experimental arm), to those of a Western-type free diet (control arm).

Secondarily, a reduction in gastrointestinal events related to chemotherapy is expected by the introduction of the MMD. As far as we know, this will be the first evaluation of the impact of the diet on chemotherapy side effects in patients with CRC, in a precision nutrition trial. We expect that improvements in therapy tolerability may also have implications in terms of treatment efficacy, by reducing dose delay, nutritional decay following gastrointestinal events, and therapy discontinuation. Our exploration of possibly impacting clinical outcomes by improving chemotherapy tolerability with an appropriate diet may generate hypotheses for future proper powered studies.

Metagenomic and metabolomic analyses will provide the identification of the metabolites produced by the GM in response to either the Mediterranean diet or the free Western diet. This knowledge could be the basis for further studies aiming at identifying the optimized diet for patients with metastatic CRC when undergoing chemotherapy.

Novel insights are expected in mechanisms and pathways linking the GM to CRC progression, and response to therapy. By investigating the impact of the Mediterranean diet on chemotherapy side effects, this study paves the way for future research on the relationship between diet-induced GM changes and therapeutic outcomes, immunotherapy responses, in patients with CRC, highlighting dietary interventions that could enhance adherence to treatment, and thus resulting in improved clinical outcomes. Specifically, the control of gastrointestinal toxicity by the targeted dietary intervention might reduce the risk of treatment delay, interruption, or dose reduction.

As highlighted in a recent review published in Science, one of the most striking findings distinguishing responders from non-responders to anti–PD-1 therapy is the differential composition of the gut microbiota, particularly the ratio of beneficial to detrimental bacterial species [53]. This was further substantiated by a prospective study by Gopalakrishnan et al. conducted in 112 patients with metastatic melanoma. Using 16S rRNA gene sequencing and whole-genome shotgun metagenomics of fecal and oral samples collected before and during anti–PD-1 therapy, the authors demonstrated that the gut microbiome profoundly influences treatment efficacy. Specifically, patients with a “favorable” gut microbiota—characterized by higher diversity and an abundance of Ruminococcaceae and Faecalibacterium—showed enhanced antitumor immunity, driven by improved antigen presentation and effector T cell activity both peripherally and within the tumor microenvironment. Conversely, those with an “unfavorable” microbiome, dominated by Bacteroidales, had a blunted systemic and intratumoral immune response with impaired lymphoid and myeloid infiltration and decreased antigen presentation [54].

This concept of microbial influence on treatment efficacy extends beyond immunotherapy. In a recent exploratory, prospective, cross-sectional study of 14 breast cancer patients treated with CDK4/6 inhibitors, specific fecal microbiota profiles were also associated with response to therapy. Bifidobacterium longum and Ruminococcus callidus were enriched in responders, while non-responders showed higher abundance of Clostridium innocuum and Schaalia odontolytica. Importantly, patients harboring favorable bacterial species also experienced better overall survival outcomes [55].

Beyond microbial taxonomy, the role of bacterial metabolites—particularly SCFAs such as butyrate, acetate, and propionate—has been well documented. SCFAs not only promote colonic regulatory T cell (Treg) induction and interleukin-10 production to maintain intestinal barrier integrity, but also act systemically. Butyrate, for instance, directly enhances the antitumor function of CD8+ T cells, contributing to improved responses to checkpoint inhibitors such as anti–PD-1/PD-L1 and anti–CTLA-4 therapies. Crucially, SCFAs are produced through microbial fermentation of dietary fibers, underscoring the importance of diet in shaping the metabolic output of the microbiota [56].

A recently randomized phase 2 clinical trial—the “DIET Study,” presented at ASCO 2025—provides compelling support for this concept. Among 43 patients with metastatic melanoma receiving immunotherapy, those randomized to a high-fiber diet experienced significantly improved objective response rates and a marked reduction in immune-related adverse events (especially grade ≥3), compared to those on a low-fiber diet [13].

These data collectively support the rationale behind our current study. In our experimental dietary arm (Mediterranean Microbiota Diet, MMD), we have designed a fiber-focused intervention that includes foods rich in high-quality prebiotic fibers and resistant starches, while moderately reducing total fiber content compared to Livelli di Assunzione di Riferimento di Nutrienti guidelines to mitigate gastrointestinal side effects of chemotherapy. Resistant starch has been shown to enhance the abundance of Firmicutes, particularly Faecalibacterium prausnitzii, a known butyrate producer essential for maintaining intestinal health. Moreover, our dietary strategy incorporates foods rich in antioxidants (fruits, vegetables, whole grains, and legumes) to promote the growth of Akkermansia muciniphila, a key species involved in preserving the mucosal barrier and reducing lipopolysaccharide-mediated inflammation.

Therefore, our metabolomic analysis is expected to identify distinct SCFA profiles and other microbial-derived metabolites that differentiate the two dietary arms, thereby helping to determine which dietary pattern best supports antitumor immunity.

An important goal in the course of our study will be to raise awareness among patients about the importance of adhering strictly to the dietary plan as part of their therapy. To facilitate compliance, study participants are provided with flexible substitution options (eg, replacing fish with white meat) for foods they find unpalatable, and adjustments designed to maintain the therapeutic rationale of the diet.

Our group is committed to the dissemination of our research products in the field of nutritional intervention in oncologic patients. We reported findings of previous research to the medical audience in international published articles, congress posters and presentations. As well, we plan to publish the results of our pilot study. In addition, we cooperate with the “Oncologia e Cucina” Association, which supports dissemination of scientific information to lay readers by an online blog. Usable information will be communicated as soon as possible to patients and caregivers, through the Association blog, with a plain language summary of published results.

### Conclusions

In conclusion, the MIC-1 study, the first precision nutrition trial of our knowledge, may provide novel insights into the role of diet in modulating the gut microbiota and metabolome, highlighting the potential of dietary interventions to reduce chemotherapy-related toxicities and enhance the efficacy of immunotherapy through SCFA-mediated immune mechanisms in solid tumors. These findings could lay the groundwork for personalized nutritional strategies in oncology based on microbiome–metabolome–host interactions

## Abbreviations

CRC: colorectal cancer
EGFR: epidermal growth factor receptor
GDPR: General Data Protection Regulation
GM: gut microbiota
KIDMED: Mediterranean diet quality index for children and adolescents
MMD: modified Mediterranean diet
OTU: operational taxonomic unit
SCFA: short-chain fatty acids
SWD: Standard Western Diet
VEGF: vascular endothelial growth factor

## Data Availability

Data are presented in the main manuscript.
